# Effect of HIV infection on TB treatment outcomes and time to mortality in two urban hospitals in Ghana-a retrospective cohort study

**DOI:** 10.11604/pamj.2019.32.206.18673

**Published:** 2019-04-26

**Authors:** Lily Ogyiri, Margaret Lartey, Oluwayemisi Ojewale, Andrew Anthony Adjei, Awewura Kwara, Richard Mawuena Adanu, Kwasi Torpey

**Affiliations:** 1 Department of Population, Family and Reproductive Health, School of Public Health, University of Ghana, Ghana; 2 Department of Medicine, School of Medicine and Dentistry, University of Ghana, Ghana; 3 Department of Medicine, College of Medicine, University of Florida, USA

**Keywords:** Tuberculosis, TB/HIV co-infection, treatment success, mortality

## Abstract

**Introduction:**

Tuberculosis (TB) is currently causing more deaths than Human Immunodeficiency Virus (HIV) globally. Ghana as one of the 30 high burden TB/HIV countries has a high annual TB case-fatality rate of 10%. The study sought to assess the effect of HIV infection on TB treatment outcomes and assess the time to mortality after treatment onset.

**Methods:**

We conducted a review of treatment files of TB patients who were treated from January 2013 to December 2015 in two urban hospitals in the Accra Metropolis. Modified Poisson regression analysis was used to measure the association between HIV infection and TB treatment outcomes. Kaplan-Meier survival estimates were used to plot survival curves.

**Results:**

Seventy-seven percent (83/107) of HIV infected individuals had successful treatment, compared to 91.2% (382/419) treatment success among HIV non-infected individuals. The proportion of HIV-positive individuals who died was 21.5% (23/107) whilst that of HIV-negative individuals was 5.5% (23/419). Being HIV-positive increased the risk of adverse outcome relative to successful outcome by a factor of 2.89(95% CI 1.76-4.74). The total number of deaths recorded within the treatment period was 46; of which 29(63%) occurred within the first two months of TB treatment. The highest mortality rate observed was among HIV infected persons (38.6/1000 person months). Of the 107 TB/HIV co-infected patients, 4(3.7%) initiated ART during TB treatment.

**Conclusion:**

The uptake of ART in co-infected individuals in this study was very low. Measures should be put in place to improve ART coverage among persons with TB/HIV co-infection to help reduce mortality.

## Introduction

Antiretroviral therapy (ART) use in areas endemic with tuberculosis (TB) is highly beneficial but HIV treatment coverage remains suboptimal. Early initiation of ART improves survival of people living with human immunodeficiency virus (HIV), reduces the incidence of TB and delays the progression of HIV [1, 2]. In patients with TB/HIV coinfection, concurrent ART during TB treatment is also associated with improved survival among patients [3, 4]. ART suppresses viral replication; transforming HIV infection from a highly fatal to a chronic disease that can be managed with a favorable prognosis [5]. There has been more than a two-fold increase in the number of people receiving ART since 2010 in the world's most affected regions - Eastern and Southern Africa. This has made a huge contribution to the reduction in acquired immune deficiency syndrome (AIDS)-related deaths worldwide - from 1.4 million in 2010 to 940,000 in 2017 [6]. It is estimated that about 54 million deaths worldwide have been prevented by TB diagnosis and treatment between the year 2000 and 2017 [7]. Yet the TB/HIV syndemic continues to claim millions of lives each year [8]. Tuberculosis accelerates the progression of HIV infection to AIDS if left untreated [9]. Deaths from TB have fallen by 42% from the year 2000 to 2017, however, TB is still one of the leading causes of mortality in the world; now causing more deaths than HIV [7]. In 2015, about 75% of TB/HIV deaths worldwide occurred in Sub Saharan Africa, with case fatality rates varying from under 5% to over 20% among countries in the region [8]. Tuberculosis mortality rate in 2015 was 47% lower than in 1990 globally. The target was to reduce the mortality rate by 50% by the year 2015, which was achieved by the WHO Region of the Americas, South-East Asia Region, Western Pacific, Eastern Mediterranean and 11 of the high TB burden countries [10]. Latest reports suggest that the WHO African Region now has one of the fastest rates of reductions in mortality (4% per year, since 2013 to 2015); second to the WHO European Region with 5% per year in the same five years [7].

The post 2015 agenda of the WHO is to end TB by the year 2035. One of the End TB Strategy targets is to eventually decrease the number of TB deaths by 95% by the year 2035 compared to 2015 [8]. Ghana is recognized as one of the 30 countries in the world with a high burden of TB/HIV co-infection; estimated at 36 per 100,000 population annually [8]. Annual case-fatality rates in Ghana have been consistently about 10% over the past few years whilst annual rates of lost-to-follow up (LTFU) and treatment failure have remained at about 3% and 2% respectively [11]. Interventions are necessary to reduce deaths during TB treatment in order for the country to achieve the targets set in the End TB Strategy by 2035. A study among 1797 patients found no significant difference between the outcomes of treatment between HIV infected and non-infected patients whilst cure rates in TB/HIV co-infected patients were found to be significantly reduced in one retrospective cohort analysis [12, 13]. Again, the odds of death was seven times higher among HIV-infected TB patients in an Ethiopian study [14]. A Nigerian study among 1424 TB patients reported a proportionate mortality of 50.6% and a mortality rate of 37.6 per 100 person months all in the first week of TB treatment onset [15]. In another study majority of the deaths occurred in the intensive phase however, the risk of death was significantly increased among persons who were TB/HIV co-infected in the continuation phase of TB treatment [16]. Even though studies have reported that HIV infection increases the risk of adverse outcome, there are some knowledge gaps with respect to death among co-infected patients in Ghana. Questions on the timing of death and the influential factors still need to be answered as this may be important for designing strategies to reduce mortality rated in TB/HIV co-infected patients. The objectives of the study were to assess the effect of HIV infection on TB treatment outcomes and the time to mortality after the onset of TB treatment.

## Methods

**Study design:** We conducted a review of treatment files and registers of TB patients who were treated from 1^st^January 2013 to 31^st^ December 2015. A data extraction form was used to collect relevant information from TB treatment cards and registers. Study participants joined the cohort on the day TB treatment was initiated until the day any of the treatment outcomes was achieved.

**Setting:** The study was conducted in two primary health care facilities located in the Accra Metropolis in the Greater Accra Region of Ghana. The Greater Accra Region is known to have one of the highest annual TB case notification rates in the country [17] and the Accra Metropolis is the most densely populated part of the Greater Accra Region with a population of about 1,665,086 in 2010 [17, 18]. Data was collected from both hospitals in May 2017.

### Participants

**Inclusion criteria:** All TB patients 15 years and above who were given TB treatment at any time from 1^st^January 2013 to 31^st^ December 2015.

**Exclusion criteria:** Patients with multi-drug resistant TB (MDR-TB). Patients who were transferred to other clinics to continue treatment.

**Selection of study participants:** All eligible participants were recruited into the study.

### Variables

**Outcome variables:** 1) TB treatment outcomes: Successful outcome (cured, treatment completed), adverse outcome (treatment failure, died and lost-to-follow up). 2) Time period between TB treatment initiation and death.

**Exposure variable:** HIV status-HIV infected or HIV non-infected.

**Potential confounders/predictors:** Age, sex, TB type-smear positive pulmonary TB, smear negative pulmonary TB and extra pulmonary TB. History of previous TB treatment-new patients, relapse patients, treatment after failure patients and treatment after lost-to-follow up patients. Treatment-cotrimoxazole preventive therapy, anti-retroviral therapy (ART).

### Data measurement

**Definition of terms:** The following are standard World Health Organisation (WHO) definitions for the various TB treatment outcomes [**19**].

**Cured:** A pulmonary TB patient with bacteriologically confirmed TB at the beginning of treatment who was smear or culture negative in the last month of treatment and on at least one previous occasion.

**Treatment completed:** A TB patient who completed treatment without evidence of failure but with no record to show that sputum smear or culture results in the last month of treatment and on at least one previous occasion were negative either because tests were not done or because results are unavailable.

**Successful treatment outcome (treatment success):** The sum of cured and treatment completed.

**Treatment failure:** a TB patient whose sputum smear or culture is positive at month 5 or later during treatment.

**Death:** All-cause mortality during anti-TB treatment.

**Lost-to-follow up:** A TB patient for whom treatment was interrupted for two consecutive months or more. It was previously termed as 'default.'

**Treatment after failure:** Patients who have previously been treated for TB and whose treatment failed at the end of their most recent course of treatment.

**Treatment after loss to follow up:** Patients who have previously been treated for TB and were declared lost-to-follow up at the end their most recent course of treatment.

**New patients:** Patients who have never been treated for TB or have taken anti-TB drugs for less than one month in the past.

**Statistical methods:** Data were analysed using STATA 14. Out of a total of 531 eligible participants, two records found to be missing data on duration of treatment and TB treatment history were dropped from the analyses. Three patients' HIV statuses were unknown and were excluded. The number of excluded observations added up to less than 1% (5/531) of the total number of observations, hence it was assumed that data were missing completely at random. A complete case analysis of 526 observations was therefore conducted. Proportions and frequencies were calculated for binary or categorical variables. A modified Poisson regression analysis was used to measure the magnitude and strength of the association between HIV infection and TB treatment outcomes. TB treatment outcomes in this regard was categorized into a binary variable, namely successful outcome (cure or treatment complete) and Adverse Outcome (died, lost-to-follow up or treatment failure). Duration of survival was calculated from the date of initiation of TB treatment to the date of death, date patient discontinued treatment (as a result of LTFU) or date of treatment completion for patients who remained alive. The outcome of interest (event) was death in the survival analysis and patients who were event free (alive) or were lost-to-follow up were censored. Kaplan-Meier survival estimates was used to determine survival function and plot survival curves. A Log-rank test was carried out to test for equality of survivor functions between the two groups (HIV infected and HIV non-infected patients). All tests were two-tailed and statistical significance was set at 0.05.

**Ethics approval:** Ethical approval was obtained from the Ghana Health Service Ethics Review Committee; reference number: GHS-ERC: 35/12/2016 and the University of Florida Institutional Review Board; IRB number: IRB201701922. A full waiver of informed consent was granted since patients were not encountered directly in the study. Data was extracted from patient treatment folders stored in the hospitals.

## Results

**Demographic characteristics and distribution of HIV infection among study participants:** in Table 1, the distribution of demographic and clinical characteristics among HIV-positive and HIV-negative TB patients is shown. Patients within the ages of 40 to 49 years had the highest proportion of HIV infections which was 28.9% (37/128). Almost 30% (54/182) of females were HIV infected; nearly twice as much infections among males. Patients with smear-negative pulmonary TB had 37.2% (45/121) HIV infections followed by those with extra-pulmonary TB - 24.2% (16/66) and smear-positive TB which was 13.6% (46/339). Table 2 illustrates the treatment outcomes by demographic and clinical profiles. Age was almost equally distributed across all the four age groups; the lowest proportion being 23.6% among 15 to 29 years and the highest being 27.8% among 50 years and over. About two-thirds of the participants were males (n = 344; 65.4%) and more than 60% of the participants were diagnosed with smear-positive TB. The overall prevalence of HIV among the participants was 20.3% (107/526) and nearly all (96%; 505/526) the patients were treated as new TB patients.

**Table 1:** Distribution of demographic and clinical characteristics among HIV infected and HIV non-infected TB patients in two urban hospitals in the Accra Metropolis

Variable	HIV infected n (%)	HIV non-infected n (%)
**Hospital**		
1^st^ hospital	43 (19.1)	182 (80.9)
2^nd^ hospital	64 (21.3)	237 (78.7)
**Sex**		
Male	53 (15.4)	291 (84.6)
Female	54 (29.7)	128 (70.3)
**Age (years)**		
15 – 29	9 (7.3)	115 (92.7)
30 – 39	35 (27.3)	93 (72.7)
40 – 49	37 (28.9)	91 (71.1)
50+	26 (17.8)	120 (82.2)
**TB type**		
PTB positive	46 (13.6)	293 (86.4)
PTB negative	45 (37.2)	76 (62.8)
Extra pulmonary TB	16 (24.2)	50 (75.8)
**TB history**		
New	103 (20.4)	401 (79.6)
Relapse	2 (14.3)	12 (85.7)
Treatment after failure	1 (16.7)	5 (83.3)
Treatment after LTFU	0 (0)	1 (100.0)
**Treatment Outcome**		
Cure	34 (31.8)	262 (62.5)
Treatment complete	49 (45.8)	120 (28.6)
Died	23 (21.5)	23 (5.5)
Lost-to-follow up	0 (0.0)	10 (2.4)
Treatment failed	1 (0.9)	4 (1.0)
**Co-trimoxazole preventive therapy**		
Yes	94 (87.0)	-
No	14 (13.0)	-
**Anti-retroviral therapy**		
Yes	4 (3.7()	-
No	103 (96.3)	-

**Table 2:** Treatment outcomes by demographic and clinical profiles of TB patients

	Total N=526 (%)	Treatment Outcome of TB among patients		
		Treatment success n (%)	Died n (%)	Treatment failure/LTFU n (%)
**Age (years)**				
15 – 29	124 (23.6)	116 (93.6)	2 (1.6)	6 (4.8)
30 – 39	128 (24.3)	116 (90.6)	11 (8.6)	1 (0.8)
40 – 49	128 (24.3)	110 (86.0)	14 (10.9)	4 (3.1)
50+	146 (27.8)	123 (84.3)	19 (13.0)	4 (2.7)
**Sex**				
Male	344 (65.4)	304 (88.4)	30 (8.7)	10 (2.9)
Female	182 (34.6)	161 (88.5)	16 (8.8)	5 (2.7)
**Hospital**				
1^st^ Hospital	225 (42.8)	201 (89.4)	12 (5.3)	12 (5.3)
2^nd^ Hospital	301 (57.2)	264 (87.7)	34 (11.3)	3 (1.0)
**TB type**				
PTB +	339 (64.5)	297 (87.6)	30 (8.9)	12 (3.5)
PTB –	121 (23.0)	107 (88.4)	12 (9.9)	2 (1.7)
Extra Pulmonary TB	66 (12.5)	61 (92.4)	4 (6.1)	1 (1.5)
**Treatment history**				
New	505 (96.0)	448 (88.7)	44 (8.7)	13 (2.6)
Retreatment	21 (4.0)	17 (81.0)	2 (9.5)	2 (9.5)
**HIV status**				
Positive	107 (20.3)	83 (77.6)	23 (21.5)	1 (0.9)
Negative	419 (79.7)	382 (91.2)	23 (5.5)	14 (3.3)

LTFU= lost-to-follow up; PTB+ =smear positive pulmonary TB; PTB – =smear negative pulmonary TB

**TB treatment outcomes:** Eighty-three out of 107 (77.6%) HIV-positive individuals had successful treatment, compared to 91.2% (382/419) treatment success among HIV-negative individuals. Thirteen percent (19/146) of those aged 50 years and above died during treatment whilst deaths among 15 to 29 year-olds was 1.6% (2/124). The proportion of HIV-positive individuals who died was 21.5% (23/107); about four times that of HIV-negative individuals - 5.5% (23/419). In the modified Poisson regression analysis illustrated in Table 3, HIV status and history of TB treatment were both significant predictors of treatment outcomes in the unadjusted analysis. In the adjusted model, only HIV status remained significant. Being HIV infected increased the risk of adverse outcome relative to successful outcome by almost 3 times (2.89; 95% CI 1.76 to 4.74). The relative risk of adverse outcome (death/treatment failure/LTFU) compared to successful outcome (cure/treatment completed) among patients who were on retreatment was not significant after controlling for sex, age, type of TB, hospital and HIV status. The same is true for all the other variables that were added to the model.

**Table 3:** Modified poisson regression (with robust standard error) of factors associated with TB treatment outcome

Predictor	Unadjusted RR (95% CI)	Adjusted RR (95% CI)
**Sex**		
Female	1	1
Male	1.00 (0.61 - 1.66)	1.16 (0.69 - 1.94)
**Age (years)**		
15 - 29	1	1
30 - 39	1.45 (0.61 - 3.44)	1.17 (0.50 - 2.72)
40 - 49	2.17 (0.98 - 4.83)	1.64 (0.70 - 3.83)
50 and over	**2.44 (1.13 - 5.27)**	2.26 (1.01 - 5.07)
**Type of TB**		
Smear positive pulmonary TB	1	1
Smear negative pulmonary TB	0.93 (0.53 - 1.65)	0.62 (0.34 -1.14)
Extra-pulmonary TB	0.61 (0.25 - 1.48)	0.54 (0.23 - 1.27)
**TB treatment history**		
New	1	1
Retreatment	**1.18 (3.08 - 4.51)**	1.61 (0.62 - 4.19)
**Hospital**		
1^st^ Hospital	1	1
2^nd^ Hospital	1.15 (0.71 - 1.87)	1.22 (0.74 - 2.03)
**HIV status**		
Negative	1	1
Positive	**2.54 (1.59 - 4.06)**	**2.89 (1.76 - 4.74)**

RR= Relative risk; TB treatment outcome= Successful outcome/ adverse outcome

**Time to deaths:** Forty-six deaths in total were recorded within the treatment period. Most of these deaths (n = 29; 63%) occurred within the first two months (intensive phase) of TB treatment as observed in Table 4. In Table 5, the highest mortality rates observed was among HIV-positive persons and persons 50 years and above; 38.6 per 1000 person months and 22.6 per 1000 person months respectively. The rate of deaths among patients in the second hospital was more than twice that in the first hospital; 19.3 per 1000 person months and 8.8 per 1000 person months respectively.

**Table 4:** Mortality since treatment onset

Time since treatment onset	Number of deaths	Proportionate mortality (%)	Person time (months)	Mortality rate per 100 pm (95% CI)
Week 1	6	13.0	1.1	541.9.4 (243.5 - 1206.3)
Week 2	5	10.9	2.7	186.7 (77.7 - 484.5)
Week 3	3	6.5	3.1	95.5 (30.8 - 296.0)
Week 4	4	8.7	3.7	107.7 (40.4 - 286.9)
2^nd^ month	11	23.9	19.8	55.7 (30.8 - 100.6)
3^rd^ month	6	13.0	17.4	34.4 (15.5 - 76.6)
4^th^ month	3	6.5	15.9	18.9 (6.1 - 58.5)
5^th^ month	3	6.5	82.2	3.6 (1.2 - 11.3)
6^th^ month+	5	10.9	2982.4	0.2 (0.1 - 0.4)

pm = person months

**Table 5:** Mortality rates across demographic and clinical variables

Variable	Number at risk	Number of deaths	Person time (months)	Mortality rate per 1000 pm (95% CI)
**Age (years)**				
15 - 29	124	2	768.9	2.6 (0.6 - 10.4)
30 - 39	128	11	762.1	14.4 (8.0 - 26.1)
40 - 49	128	14	757.5	5.3 (10.9 - 31.2)
50 and above	146	19	839.7	22.6 (14.4 - 35.5)
**Sex**				
Male	344	30	2058.5	14.6 (10.2 - 20.8)
Female	182	16	1069.8	14.9 (9.2 - 24.4)
**Type of TB**				
Smear positive TB	339	30	1987.1	15.1 (10.5 - 21.6)
Smear negative TB	121	12	680.1	17.6 (10.0 - 31.1)
Extra-pulmonary TB	66	4	461.0	8.7 (3.3 - 23.1)
**TB treatment history**				
New	505	44	2977.2	14.8 (11.0 - 19.9)
Retreatment	21	2	151.1	13.2 (3.3 - 52.9)
**HIV status**				
Positive	107	23	596.0	38.6 (25.6 - 58.1)
Negative	419	23	2532.3	9.1 (6.0 - 13.7)
**Hospital**				
1^st^ hospital	225	12	1370.4	8.8 (5.0 - 15.4)
2^nd^ hospital	301	34	1757.9	19.3 (13.8 - 27.1)
**Year**				
2013	191	14	1165.7	12.0 (7.1 - 20.3)
2014	156	14	905.9	15.5 (9.2 - 26.1)
2015	179	18	1056.7	17.0 (10.7 - 27.0)

pm = person months

## Discussion

The probability of survival at the end of the analysis time among the total sample of patients in this study was 89.4%. Plotting the curves separately according to HIV status showed that those with HIV infection had a lower survival, confirming documented evidence that being HIV infection increases one's risk of mortality during TB treatment. Our finding of higher mortality in TB/HIV co-infected patients compared to those with TB only is similar to that reported among patients attending a chest clinic in a teaching hospital in Ghana, but in contrast, we did not find a difference in default rates between the two groups [20]. Again, in Sudan, a significantly higher case-fatality rate among persons with TB/HIV co-infection (12%) compared to those with TB infection only (1.8%) was found. HIV tests were conducted among patients in the study however, results were anonymised and not linked [13]. The proportion of LTFU and treatment failure which is less than 3% of the entire cohort corroborates with previous reports about TB treatment outcomes in other countries and in Ghana [11, 13]. The component of adverse outcome which was most influenced or affected by HIV infection was death. Interventions targeted towards reducing deaths among TB patients may help contribute to End TB Strategy goals and targets. The effect of the concurrent use of anti-TB treatment and ART could not be estimated in this study because only four (3.8%) were documented to have been given concurrent treatment and hence could not be included in the analyses. It is quite surprising to see that such a low proportion of HIV infected individuals in this study were given concurrent ART. In a study in Nigeria, 16% out of 568 TB/HIV co-infected patients received concurrent treatment. Survival was lowest among those who were not on ART and their risk of mortality was aHR 1.39 (95 %CI 1.04 to 1.86) compared to those who were on ART [15]. The low uptake of ART in our study may have contributed to the high mortality among the HIV co-infected patients since several studies show that integrated therapy has significant survival benefit [21-23]. One retrospective study on the predictors of mortality among TB/HIV co-infected patients reported a significant increase in the risk of mortality (aHR = 3.15 95%CI 1.95 to 5.11) among patients who were not given cotrimoxazole preventive therapy during treatment. They also found that patients on antiretroviral therapy (ART) had a hazard ratio of 0.35 (95%CI 0.19 to 0.64) [24]. Agreeably, HIV patients in this study who were not started on cotrimoxazole preventive therapy during treatment had a hazard ratio of 4.18 (95%CI 1.71 to 10.21). This report is also consistent with that of a study conducted in Cameroun [25].

Additionally, according to two other studies, patients who do not take cotrimoxazole preventive therapy have an increased risk of death or adverse outcome [26, 27]. It is estimated that about 25% of TB/HIV co-infected people in Ghana receive concurrent treatment despite recommendations in the WHO guidelines for TB/HIV co-infection. ART should be initiated for all patients with TB/HIV co-infection regardless of their CD4 cell count. TB treatment should be started first, followed by ART as soon as possible and within the first eight weeks of starting TB treatment [19]. There is a need for qualitative studies to bring out the challenges or barriers to the implementation of the guidelines especially in the Ghanaian setting. Possible solutions to this problem may include training health professionals in concurrent management of TB and HIV or to develop optimal models to facilitate integration of TB and HIV services in health facilities. The difference in the mortality rates between the two hospitals in this study may have been due to institutional factors, patient-factors or both. The rate of death among the HIV-positive persons in this study was almost twice (38.6 per 1000 person months) that found in an Ethiopian study where among TB/HIV co-infected patients, a rate of 20.6 per 1000 person months was recorded. However, the mortality rates among HIV non-infected persons were similar in both studies; 10.8 per 1000 person months in the Ethiopian study and 9.1 per 1000 person months in our study [16]. Most studies report that majority of these deaths occur in the first few weeks of treatment [15, 28]. In our study, the proportionate mortality at the end of the first two weeks was 23.9% and 63.0% at the end of the first two months. A possible explanation to this observation is that those who die in the first few weeks of treatment report to the hospitals late. The causes of delay may be due to fear of drug toxicities, stigmatisation, self-medication, delay in diagnosis of TB and/or HIV infection and counselling among other others [8, 29].

**Limitations of the study:** Missing data on variables such as weight and height made it impossible to test their associations with the outcomes. Also, information on CD4 cell counts, drug adherence, co-morbidities and other socio demographic variables that could have predicted outcomes could not be analysed because they were not available.

## Conclusion

HIV infection significantly increased the risk of death among the patients. More than 60% of deaths occurred in the first two months of TB treatment. Initiation of ART within eight weeks of starting anti-TB treatment as recommended by WHO [28] may have averted some of these deaths. Measures should be put in place to improve ART coverage among persons with TB/HIV co-infection to help reduce mortality.

### What is known about this topic

- HIV infection increases the risk of death among TB patients;
- Administration of concurrent ART and anti-TB therapy reduces the risk of death among TB/HIV co-infected patients.

### What this study adds

- The co-administration of ART and anti-TB therapy is suboptimal in some hospitals in the Accra metropolis;
- Health system factors in some hospitals in the Accra metropolis may influence mortality among TB patients.

## Competing interests

The authors declare no competing interests.

## References

[1] Temprano ANRS 12136 Study Group (2015). A Trial of Early Antiretrovirals and Isoniazid Preventive Therapy in Africa. New Engl J M.

[2] Grinsztejn B, Hosseinipour MC, Ribaudo HJ, Swindells S, Eron J, Chen YQ (2014). Effects of early versus delayed initiation of antiretroviral treatment on clinical outcomes of HIV-1 infection: results from the phase 3 HPTN 052 randomised controlled trial. Lancet Infect Dis.

[3] Mutembo S, Mutanga JN, Musokotwane K, Alisheke L, Whalen CC (2016). Antiretroviral therapy improves survival among TB-HIV co-infected patients who have CD4 + T-cell count above 350cells. BMC Infect Dis.

[4] Franke MF, Robins JM, Mugabo J, Kaigamba F, Cain LE, Julia G (2011). Effectiveness of Early Antiretroviral Therapy Initiation to Improve Survival among HIV-Infected Adults with Tuberculosis: A Retrospective Cohort Study. PLoS Med.

[5] Maartens G, Celum C, Lewin SR (2014). HIV infection: epidemiology, pathogenesis, treatment, and prevention. Lancet.

[6] UNAIDS Global HIV & AIDS statistics-2018 fact sheet.

[7] WHO (2018). Global Tuberculosis Report 2018.

[8] WHO (2016). Global Tuberculosis Report 2016.

[9] Mayer KH, Dukes Hamilton C (2010). Synergistic pandemics: confronting the global HIV and tuberculosis epidemics. Clin Infect Dis.

[10] WHO (2015). Global tuberculosis report 2015.

[11] GHS (2015). Ghana Health Service 2014 Annual Report.

[12] Ambadekar NN, Zodpey SP, Soni RN, Lanjewar SP (2015). Treatment outcome and its attributes in TB-HIV co-infected patients registered under Revised National TB Control Program: a retrospective cohort analysis. Public Health.

[13] El-Sony AI, Khamis AH, Enarson DA, Baraka O, Mustafa SA, Bjune G (2002). Treatment results of DOTS in 1797 Sudanese tuberculosis patients with or without HIV co-infection. Int J Tuberc Lung Dis.

[14] Gebremariam G, Asmamaw G, Hussen M, Hailemariam MZ, Asegu D, Astatkie A (2016). Impact of HIV status on treatment outcome of tuberculosis patients registered at Arsi Negele Health Center, Southern Ethiopia: a six year retrospective study. PLoS One.

[15] Adamu AL, Gadanya MA, Abubakar IS, Jibo AM, Bello MM, Gajida AU (2017). High mortality among tuberculosis patients on treatment in Nigeria: a retrospective cohort study. BMC Infect Dis.

[16] Shaweno D, Worku A (2012). Tuberculosis treatment survival of HIV positive TB patients on directly observed treatment short-course in Southern Ethiopia: a retrospective cohort study. BMC Res Notes.

[17] Ministry of Health (2008). The National TB Health Sector Strategic Plan for Ghana, 2009-2013.

[18] Ghana Statistical Service (2014). 2010 Population & Housing Census: District analytical report, Accra Metropolitan.

[19] WHO (2010). Treatment of tuberculosis: guidelines.

[20] Burton NT, Forson A, Lurie MN, Kudzawu S, Kwarteng E, Kwara A (2011). Factors associated with mortality and default among patients with tuberculosis attending a teaching hospital clinic in Accra, Ghana. Trans R Soc Trop Med Hyg.

[21] Blanc F-X, Sok T, Laureillard D, Borand L, Rekacewicz C, Nerrienet E (2011). Earlier versus later start of antiretroviral therapy in HIV-infected adults with tuberculosis. N Engl J Med.

[22] Havlir D, Kendall MA, Ive P, Kumwenda J, Swindles S, Qasba S (2011). Timing of antiretroviral therapy for HIV-1 infection and tuberculosis. N Engl J Med.

[23] Abdool Karim SS, Naidoo K, Grobler A, Padayatchi N, Baxter C, Gray AL (2011). Integration of antiretroviral therapy with tuberculosis treatment. N Engl J Med.

[24] Sileshi B, Deyessa N, Girma B, Melese M, Suarez P (2013). Predictors of mortality among TB-HIV co-infected patients being treated for tuberculosis in Northwest Ethiopia: a retrospective cohort study. BMC Infect Dis Dis.

[25] Agbor AA, Bigna JJR, Billong SC, Tejiokem MC, Ekali GL, Plottel CS (2014). Factors associated with death during tuberculosis treatment of patients co-infected with HIV at the Yaounde Central Hospital , Cameroon: an 8-year hospital-based retrospective cohort study (2006-2013 ). PLoS One.

[26] Ali SA, Mavundla TR, Fantu R, Awoke T (2016). Outcomes of TB treatment in HIV co-infected TB patients in Ethiopia: a cross-sectional analytic study. BMC Infect Dis.

[27] Pimchan N, Suggaravetsiri P, Tesana N, Chaiklieng S (2012). The associated factors with unsuccessful tuberculosis treatment outcomes among TB/HIV co-infected patients in Surin Province, Thailand. Res J Med Sci.

[28] Ismail I, Bulgiba A (2013). Predictors of death during tuberculosis treatment in TB / HIV co-infected patients in Malaysia. PLoS One.

[29] Maponga B, Chirundu D, Gombe N, Tshimanga M, Bangure D, Takundwa L (2015). Delayed initiation of anti-retroviral therapy in TB/HIV co-infected patients, Sanyati District, Zimbabwe, 2011-2012. Pan Afr Med J.

